# Predicting a reduction in intraocular pressure in glaucoma patients in the early period after a trabeculectomy: Development and assessment of a new predictive nomogram

**DOI:** 10.3389/fopht.2022.987742

**Published:** 2022-10-27

**Authors:** Ruixue Wang, Ning Li, Yue Tan, Xiaoya Chen

**Affiliations:** ^1^ Dapartment of Ophthalmology, The Affiliated Xuzhou Municipal Hospital of Xuzhou Medical University, Xuzhou, China; ^2^ Dapartment of Ophthalmology, Xuzhou First People’s Hospital, Ophthalmology, Xuzhou, China; ^3^ Department of Ophthalmology, the first Affiliated Hospital of Anhui Medical University, Hefei, China

**Keywords:** glaucoma, trabeculectomy, intraocular pressure, nomogram, predictor

## Abstract

**Purpose:**

To identify the factors associated with a reduction in intraocular pressure (IOP) in the early postoperative period after a trabeculectomy and to develop a predictive nomogram to guide clinical care.

**Methods:**

This study included clinical data on 588 glaucoma patients (*N* = 588 eyes) who underwent a trabeculectomy in our hospital between January 2016 and December 2021. There were 412 eyes in a training cohort and 176 eyes in a validation cohort. We used logistic regression analysis to evaluate whether these factors were related to a decrease in IOP in the early period postsurgery and established a predictive model by combining features selected in a univariate analysis. We used external validation for evaluation. The standard for IOP reduction was that the IOP decreased to the normal range (10−21 mmHg) 1 month after the trabeculectomy.

**Results:**

Among the patients in the training cohort, 82.8% met the standard for IOP lowering. There were 11 meaningful differences among the enrolled predictors, but the logistic regression analysis only showed significant differences with anterior chamber angle closed, age, preoperative IOP, axial length, and visual field mean sensitivity (MS). The C-index of the model was 0.910 (95% confidence interval [CI]: 0.869-0.951). The *C*-index was 0.956 for external validation of the model.

**Conclusion:**

This new nomogram can be used to predict whether the IOP will reach the standard in the early stages after a trabeculectomy. The anterior chamber angle closed, age, preoperative IOP, axial length, and visual field MS are independent risk factors.

## Introduction

Good vision is a basic requirement for good quality of life. Glaucoma, as the primary cause of irreversible blindness worldwide, poses a major threat to visual function. Although glaucoma can grow blind, it can also be prevented and treated. Regular screening, early diagnosis and treatment can slow the progression of the disease. However, a considerable number of patients are already in the middle and late stages when the disease is discovered. Thus, effective treatment strategies are vital (1, 2).

It is well known that pathological elevated intraocular pressure (IOP) is a significant risk factor for glaucoma progression (3, 4). At present, reducing IOP remains the most important treatment option for relieving glaucoma (5, 6). Keeping the IOP stable in the normal range can delay the rate of vision loss to some extent (5, 6). The main surgical way to reduce IOP is a trabeculectomy. As a classic surgery for the treatment of glaucoma, a trabeculectomy is effective, and it has been widely used to treat various types of glaucoma since it was first proposed in 1968 (7). In trabeculectomy, the limbus is used as the base to make bulbar conjunctival flap and scleral flap to create a new extraocular drainage channel to drain the aqueous humor produced by the ciliary body from the anterior chamber to the subconjunctival space, and then to absorbed by surrounding tissues, thereby reducing IOP. At the same time, the scleral flap made during the operation can cover the drainage port, limit the excessive outflow of aqueous humor, and reduce the incidence of postoperative low IOP and shallow anterior chamber (8–10). However, in some patients, the surgical outcomes are unsatisfactory, with no decrease in the IOP dose or no decrease in IOP values to the normal range after the operation.

The aim of this study was to identify risk factors associated with early IOP reduction after trabeculectomy in patients with glaucoma and to develop a predictive nomogram with the hope of providing a rationale for the selection of glaucoma patients requiring surgery.

## Patients and methods

### Patient selection

**Figure 1:**
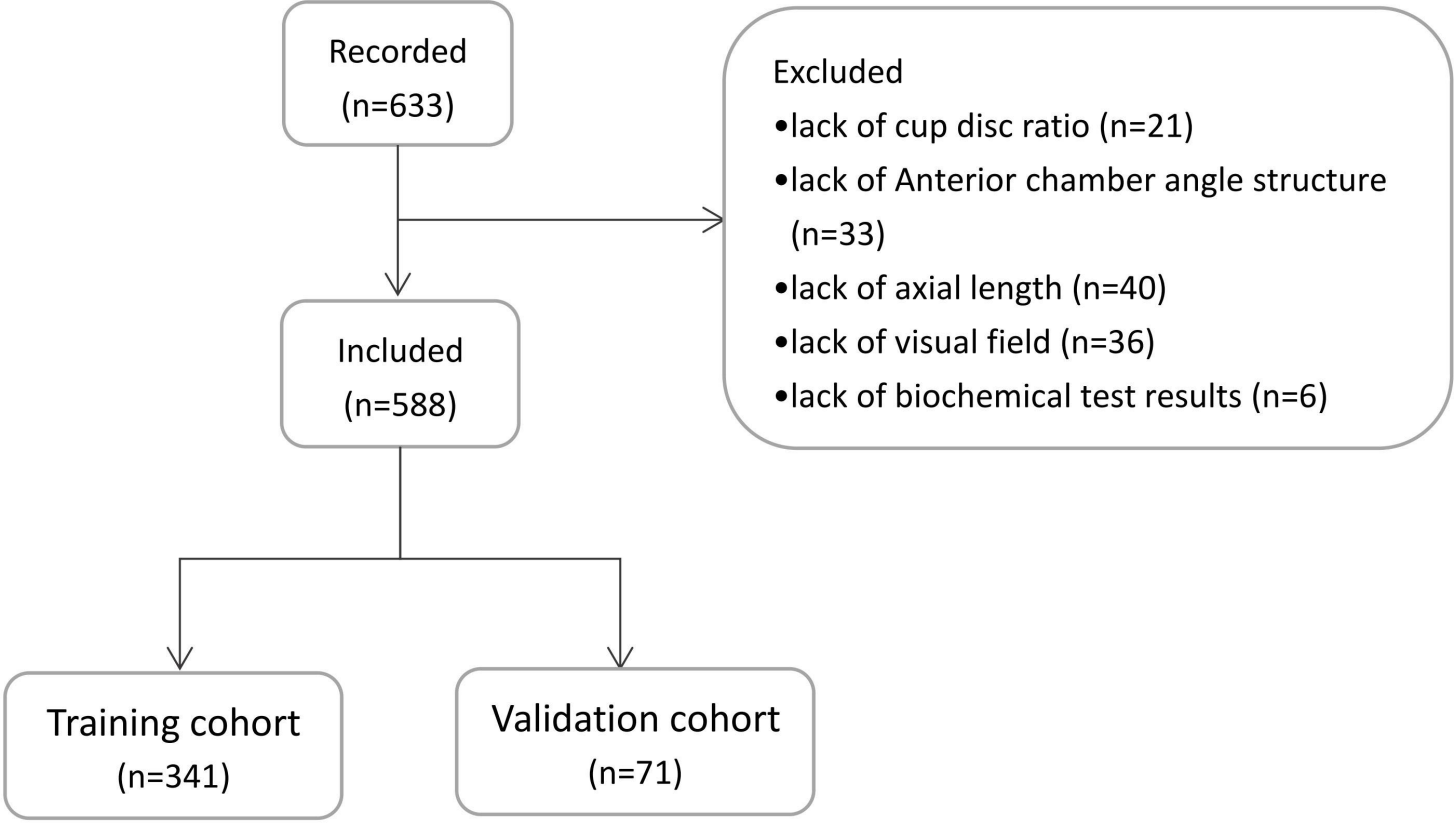
Schematic diagram of the population selection.

We used data on 443 glaucoma patients (445 eyes) who underwent a trabeculectomy in Ophthalmology Department of the Xuzhou No.1 Peoples Hospital and the First Affiliated Hospital of Anhui Medical University between January 2016 and December 2019 to establish a predictive model. From January 2020 to December 2021, we recorded clinical data on 188 patients (188 eyes) for external validation of the predictive model. Among the 633 eyes included in the study, 45 eyes were excluded. Among these, 21 eyes lacked cup disc ratio data, 33 eyes lacked anterior chamber angle structure data, 40 eyes lacked axial length data, 36 eyes lacked visual field results, and 6 eyes lacked biochemical test results. A total of 588 patients (588 eyes) were included in the final study. A schematic diagram of the population selection is shown in **Figure 1**.

This study was approved by the ethics committees for human research of the Xuzhou No.1 Peoples Hospital and the First Affiliated Hospital of Anhui Medical University. All experiments were in accordance with the relevant guidelines and the Declaration of Helsinki.

### Clinical characteristics

We extracted relevant clinical characteristics of the selected patients as potential predictors, with a total of 17 items selected. For the continuous variables, such as age, preoperative IOP, axial length, visual field mean sensitivity (MS), visual field mean defect (MD), lymphocyte and globulin ratio, the difference was compared by directly calculating the mean and standard deviation, and the other features were grouped and analyzed according to the situation. We divided glaucoma into refractory and nonrefractory to understand whether different types have different manifestations after surgery (11). Optic disc parameters, including the cup disc ratio, were measured using optical coherence tomography (12, 13). Meanwhile, with the maturity and popularization of intraocular lens implantation, intraocular lenses have become common. An intraocular lens was regarded as one of the predictors. All the patients underwent a comprehensive examination by an experienced ophthalmologist, including an evaluation of the anterior chamber angle, lens, vitreous body and iris.

### Evaluation standard

A trabeculectomy mainly reduces the IOP of patients to slow down optic nerve damage in glaucoma. The results of IOP were used as the prediction target and grouping basis in this study. The evaluation standard for IOP was an IOP value in the normal range (10−21 mmHg) 1 month after the surgery. Eyes that met the standard were defined as effective, and eyes that did not meet the standard were defined as ineffective.

### Statistics

For statistical analysis, IBM SPSS Statistics for Windows, version 23.0 (IBM Corp., Armonk, N.Y., USA) and R package, version 4.0.2 (https://www.r-project.org) were used. The nomogram was developed using the library “rms” in R for Windows. According to the website tutorial, use https://www.mskcc.org/departments/epidemiologybiostatistics/health-outcomes/decision-curve-analysis-01 for DCA analysis. The reported statistical tests were all two-sided, statistical significance was accepted as P < 0.05.

The distribution of the clinical characteristics of the selected patients is shown as the percentage, mean, or standard deviation. Univariate and multivariate regression analyses were used to filter indicators. The measurement data are expressed as mean and standard deviation. A t-test was used for between-group comparisons. Enumeration data are expressed as percentages, and a chi-square test was used for comparisons between groups. By combining the selected features of the univariate analysis, independent risk factors were screened using multivariate logistic regression analysis, weighted by their respective coefficients (14). These features were regarded as the odds ratio (OR) with 95% confidence interval (CI) and as P-value. The predictive model included sociodemographic variables and disease-related characteristics significantly associated with what exactly (P < 0.05). Based on the cohort research results from multivariate analysis, we constructed a predictive model integrating five clinical risk factors, all the potential predictors were used to develop a nomogram.

To quantify the recognition performance of the nomogram, we measured Harrell’s C-index and performed external validation of the nomogram to obtain the relatively corrected C-index (14–16). Calibration curves were drawn in predicting the likelihood of IOP lowering postsurgery (17, 18). The calibration curve shows the relationship between the predicted and the observed risk for each variable. Therefore, the ideal nomogram should exactly fits the 45-degree reference line. To determine the clinical value of the nomogram, we used decision curve analysis (DCA) to quantify the relationship between benefit and risk (i.e., net benefit) under different threshold probabilities in the study cohort (19, 20). The net benefit was used to weigh the pros and cons of missing the intervention and avoiding an unnecessary intervention. The net benefit was calculated by subtracting the proportion of false-positive cases from the proportion of true-positive cases. This method of evaluating predictive models not only shows the discriminative accuracy of the nomogram, but also visualizes the clinical effects of surgery.

## Results

### Patients’ characteristics

**Figure 2:**
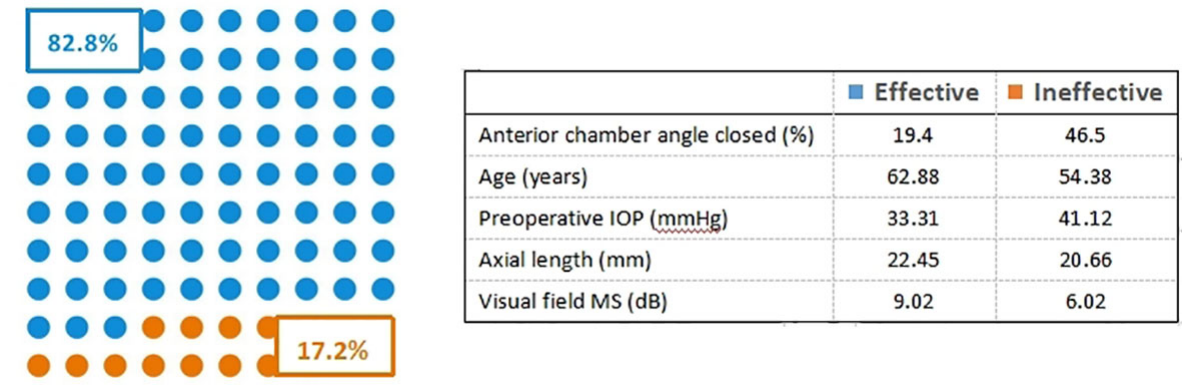
Distribution of predictors among effective and ineffective cohorts.

We divided the collected 588 patients (588 eyes) into a training cohort of 412 patients (412 eyes) and a validation cohort of 176 eyes. The training cohort is used to screen predictors and build predictive models. The validation cohort is used to verify the accuracy of the model. Meanwhile, according to the evaluation criteria for IOP, we divided the two cohorts of patients into effective and ineffective cohorts. In the training cohort, there were 341 (82.8%) patients in the effective cohort and 71 (17.2%) patients in the ineffective cohort, as shown in **Figure 2**. The validation cohort included 151 patients (85.8%) in the effective cohort and 25 (14.2%) patients in the ineffective cohort. The demographic and disease characteristics of the two cohorts of patients are shown in **Table 1**.

**Table 1:** Differences between demographic and clinical characteristics of training and validation cohorts.

Demographic characteristics	Training cohort (n = 412)				Validation cohort (n = 176)	
	Effective (n = 341)	Ineffective (n = 71)	Total	P-value	Effective (n = 151)	Ineffective (n = 25)
**n (%)**						
Cup disc ratio				0.007*		
≤0.6	83 (24.3)	7 (9.9)	90 (21.8)		31 (20.5)	1 (4.0)
>0.6	258 (75.7)	64 (90.1)	322 (78.2)		120 (79.5)	24 (96.0)
Course of disease				0.044*		
acute	221 (64.8)	37 (52.1)	258 (62.6)		90 (59.6)	13 (52.0)
chronic	120 (35.2)	34 (47.9)	154 (37.4)		61 (40.3)	12 (48.0)
Anterior chamber angle closed				0.000*		
Yes	66 (19.4)	33 (46.5)	99 (24.0)		30 (19.9)	14 (56.0)
No	275 (80.6)	38 (53.5)	313 (76.0)		121 (80.1)	11 (44.0)
Lens opacity				0.010*		
Yes	277 (81.2)	48 (67.6)	325 (78.9)		130 (86.1)	16 (64.0)
No	64 (18.8)	23 (32.4)	87 (21.1)		21 (13.9)	9 (36.0)
Refractory glaucoma				0.147		
Yes	141 (41.3)	36 (50.7)	177 (43.0)		56 (37.1)	15 (60.0)
No	200 (58.7)	35 (49.3)	235 (57.0)		95 (62.9)	10 (40.0)
Pseudophakic				0.130		
Yes	11 (3.2)	5 (7.0)	16 (3.9)		4 (2.6)	2 (8.0)
No	330 (96.8)	66 (93.0)	396 (96.1)		147 (97.4)	23 (92.0)
Sex				0.014*		
Male	138 (40.5)	40 (56.3)	178 (43.2)		64 (42.4)	14 (56.0)
Female	203 (59.5)	31 (43.7)	234 (56.8)		87 (57.6)	11 (44.0)
Binocular				0.119		
Yes	193 (56.6)	33 (46.5)	226 (54.9)		74 (49.0)	11 (44.0)
No	148 (43.4)	38 (53.5)	186 (45.1)		77 (51.0)	14 (56.0)
Vitreous opacity				0.547		
Yes	252 (73.9)	50 (70.4)	302 (73.3)		120 (79.5)	16 (64.0)
No	89 (26.1)	21 (29.6)	110 (26.7)		31 (20.5)	9 (36.0)
Unclear iris texture				0.147		
Yes	152 (44.6)	25 (35.2)	177 (43.0)		69 (45.7)	11 (44.0)
No	189 (55.4)	46 (64.8)	235 (57.0)		82 (54.3)	14 (56.0)
**Mean (SD)**						
Age (years)	62.88 (10.88)	54.38 (15.03)	61.41 (12.14)	0.000*	63.16 (10.98)	54.24 (17.22)
Preoperative IOP (mmHg)	33.31 (12.60)	41.12 (12.57)	34.66 (12.94)	0.000*	34.46 (13.30)	37.04 (8.02)
Axial length (mm)	22.45 (1.02)	20.66 (1.31)	22.14 (1.27)	0.000*	22.38 (0.96)	20.38 (1.11)
Visual field MS (dB)	9.02 (7.63)	6.02 (5.14)	8.50 (7.35)	0.000*	8.60 (6.41)	6.18 (5.62)
Visual field MD (dB)	18.05 (7.09)	20.78 (5.29)	18.52 (6.89)	0.000*	18.03 (6.57)	20.28 (6.13)
Lymphocyte (10^9^/L)	1.82 (0.61)	1.92 (0.68)	1.84 (0.62)	0.224	1.78 (0.55)	2.10 (1.18)
Globulin ratio	1.71 (0.32)	1.81 (0.31)	1.73 (0.32)	0.019*	1.69 (0.30)	1.64 (0.31)

IOP, intraocular pressure; MS, mean sensitivity; MD, mean defect. *P<0.05.

### Selection of predictors

Based on the univariate analysis, we screened 11 related risk factors from the initial 17 variables, and then excluded the influence of other confounding factors through logistic regression analysis, and finally selected 5 predictors (anterior chamber angle closed, age, preoperative IOP, axial length, and visual field MS) to build a predictive model. Excluding other parameters, with a ratio of about 3:1, as shown in **Figure 2**. **Table 2** shows the regression coefficients and ORs, together with the 95% CIs and *P*-values for these predictors.

**Table 2:** Predictors for IOP in the early postoperative period after a trabeculectomy.

Intercept and variable	Prediction model		
	β	Odds ratio (95% CI)	P-value
Anterior chamber angle closed	1.2122	3.361 (1.534 - 7.477)	0.00255
Age	-0.0390	0.962 (0.929 - 0.994)	0.02328
Preoperative IOP	0.0475	1.049 (1.017 - 1.084)	0.00355
Axial length	-1.4947	0.224 (0.148 - 0.322)	<0.001
Visual field MS	-0.1448	0.865 (0.751 - 0.988)	0.03405

IOP, intraocular pressure; MS, mean sensitivity.

### Development of the predictive nomogram

**Figure 3:**
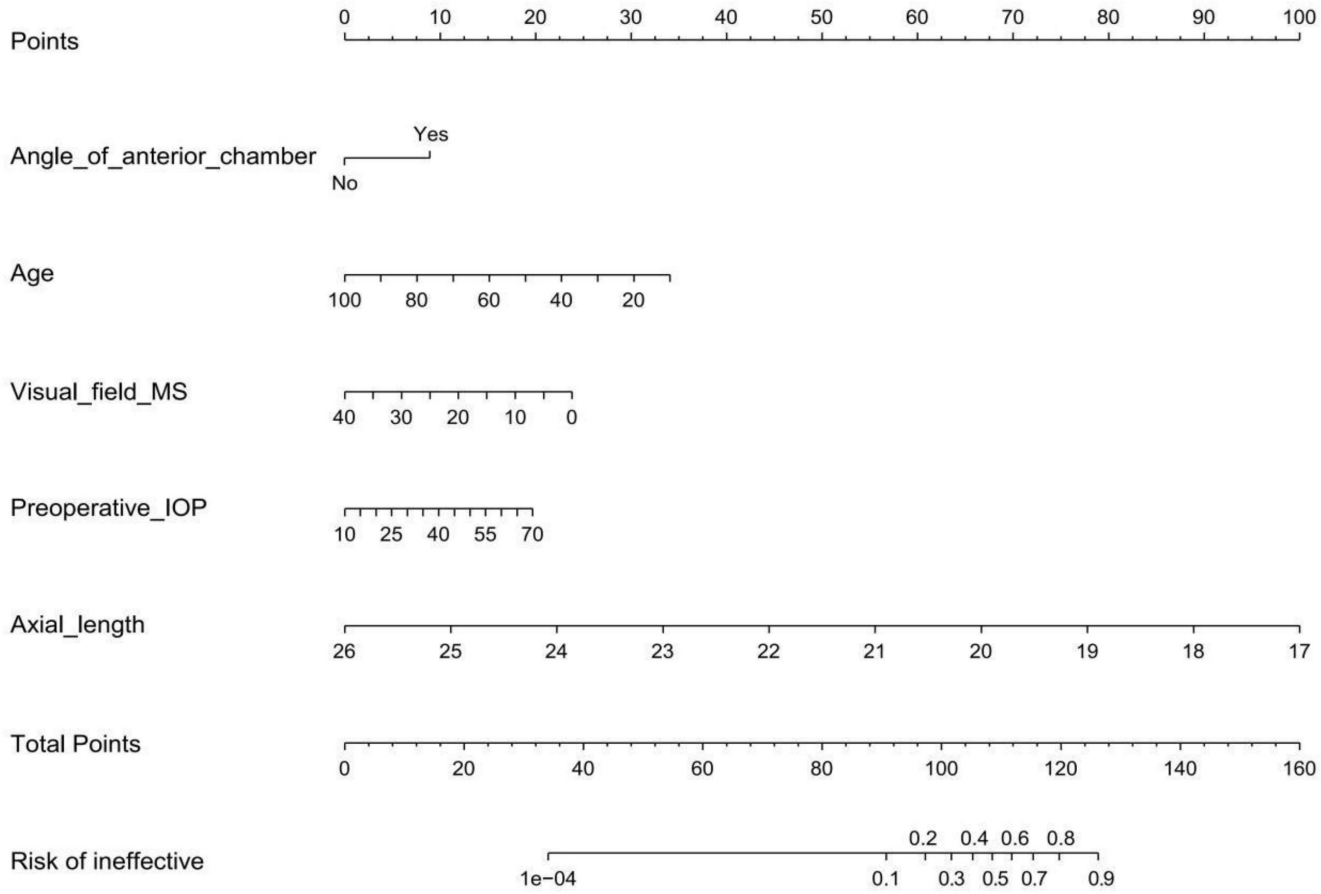
Developed a trabeculectomy nomogram.

To develop an individualized prediction model for IOP lowering in the early period after a trabeculectomy, according to the logistic regression analysis results shown in **Table 2**, we established a model containing five independent risk factors: anterior chamber angle closed, age, preoperative IOP, axial length, and visual field MS. In the selected population, a nomogram composed of the five predictors was constructed, and the risk probability was calculated using the R package, as shown in **Figure 3**. The nomogram can be explained by a summary of the points assigned to each predictor, which are represented at the top of the scale. These total scores can be converted into the risk of ineffective events occurring.

### Validation and calibration

**Figure 4:**
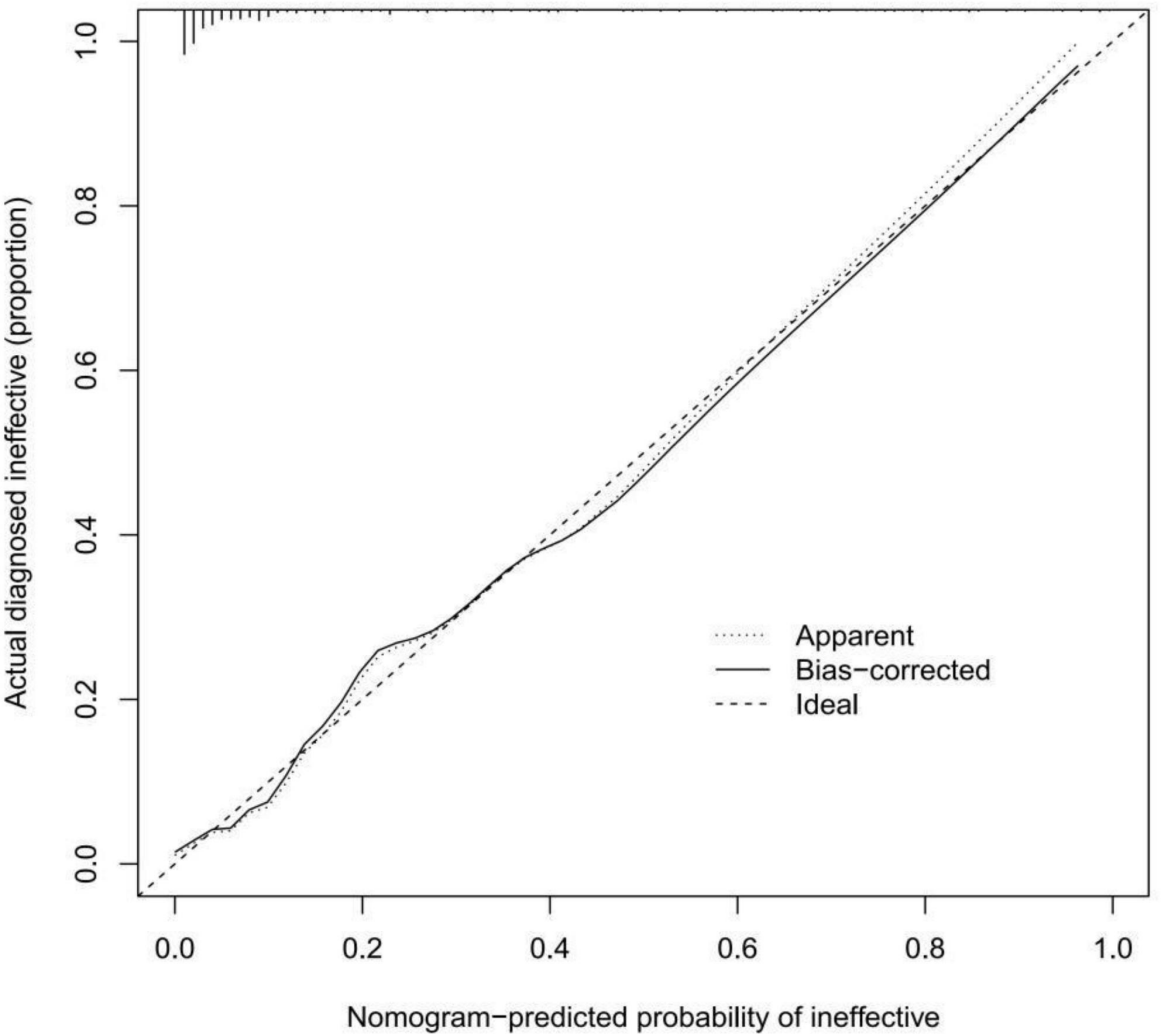
Calibration curves of a trabeculectomy nomogram prediction in the cohort.

A calibration curve of the prediction model was drawn to illustrate the relationship between the prediction probability and the actual probability. **Figure 4** shows that the training cohort and validation cohort have high goodness of fit. In addition, the discrimination of the prediction model was ideal, with a C-index of 0.910 (95% CI: 0.869-0.951). The C-index for external validation of the model was high (0.956, 95% CI: 0.914-0.998).

### Clinical use

**Figure 5:**
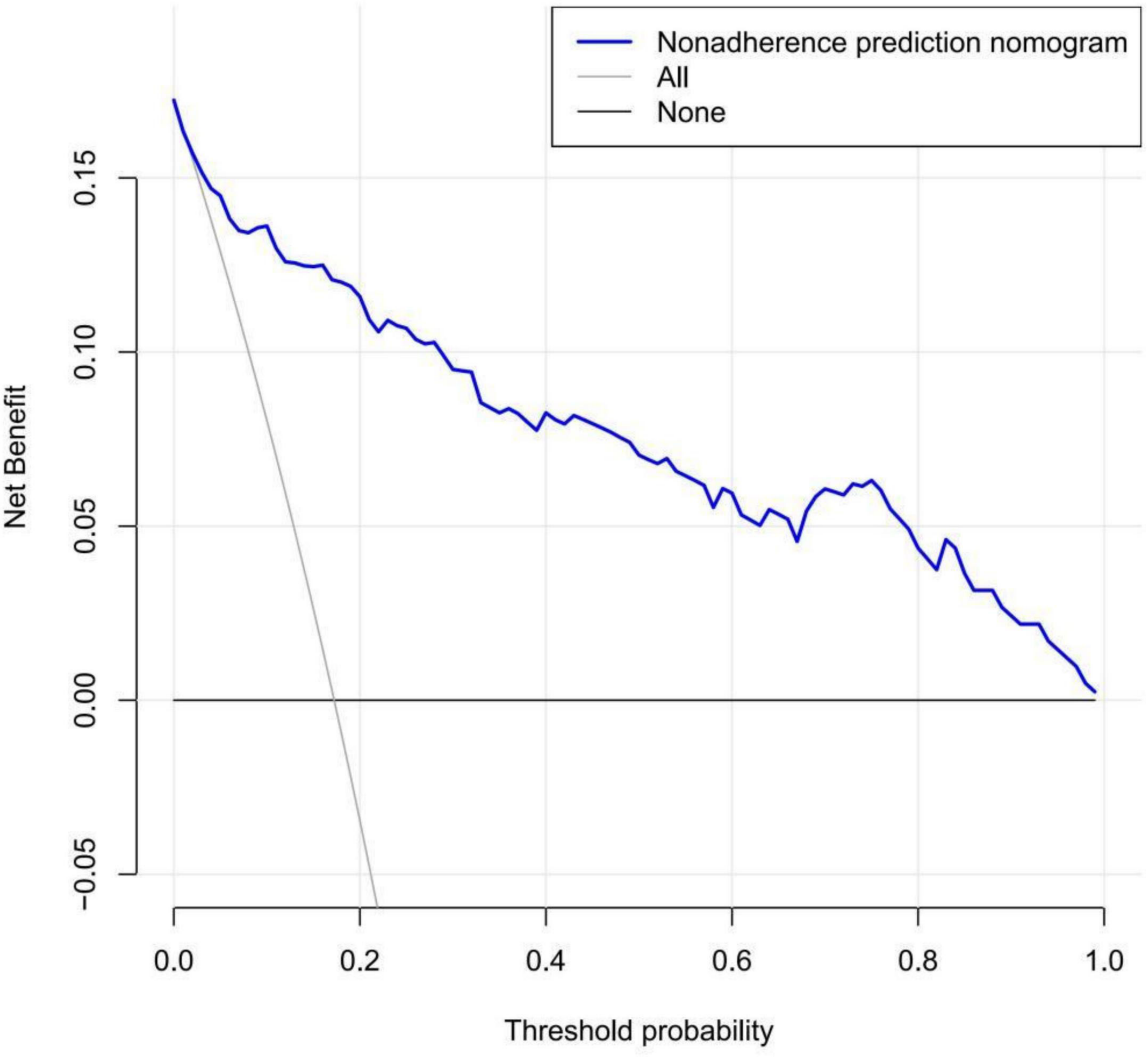
Decision curve for a trabeculectomy nomogram.

We used DCA to determine the clinical utility of the prediction model and quantify the net benefits of different threshold probabilities in the data set. **Figure 5** shows the results of the DCA of the effect of a reduction in IOP in the early period postsurgery. The decision curve shows that when the threshold probability of patients is greater than 1%, it is more beneficial to use this nomogram to predict the risk of a substandard IOP reduction in the early postoperative period than the all patients surgery scheme or the no patients surgery scheme. Within this range, the net benefit was comparable with several overlaps, on the basis of the IOP prediction nomogram (21).

## Discussion

Nomograms are widely used to aid clinical decision making in medicine and oncology (22, 23). The scope of nomograms continues to expand, and research on the use of nomograms for predicting surgical outcomes is increasing. By integrating different prognostic and determinant variables, a nomogram can be used to construct a personalized clinical model (17, 24). In this study, we combined a nomogram with the prognosis of a trabeculectomy to predict IOP lowering in the early postoperative period.

The primary purpose of a trabeculectomy is to reduce IOP. It is important to be able to accurately predict the probability of achieving IOP lowering postsurgery. In the present study, with the goal of providing guidance for clinicians, we selected potential predictors of IOP lowering in the early period after a trabeculectomy (5, 25). As shown by our analysis, five factors were significantly associated with early postoperative IOP in the patient population: anterior chamber angle closed, age, preoperative IOP, axial length, and visual field MS. These five factors may play a key role in the probability of IOP lowering postsurgery.

For glaucoma, especially primary angle-closure glaucoma, the anterior chamber angle has a major influence on the state of an illness (26, 27). Upon completion of peripheral iridectomy, the anterior and posterior chambers can communicate, and the aqueous humor flows smoothly. However, if the anterior chamber angle is completely closed, the forward flow of aqueous humor is blocked, leading to reverse flow. As a result, IOP lowering may not be achieved. Age is an independent risk factor for surgical success in terms of IOP lowering postsurgery. As shown in **Table 1**, younger individuals have a higher risk of failing to achieve the standard postoperative IOP reduction effect. On the one hand, this can be explained by young individuals having a faster metabolism, higher degree of postoperative inflammation, and higher degree of ciliary body edema than older individuals, which leads to ciliary ring block, affecting the effect of lowering IOP. On the other hand, the inflammatory mediator between the ciliary body and the lens can form exudative membrane, causing poor drainage and reverse flow of the aqueous humor, and then affect IOP (11, 28). Patients with persistent high IOP account for a certain proportion of the glaucoma population. High IOP is associated with intraocular tissue damage, with the duration of elevated IOP affecting the level of damage. Visual function damage is irreversible in patients who have persistently high IOP before surgery, and the possibility of postoperative fundus hemorrhage is increased (28). The IOP of such patients is unlikely to be reduced as scheduled (28).

In addition, axial length was closely associated with postoperative IOP in the present study. Axial length is related to the position of the lens. When aqueous humor is drained from the eye during surgery, the position of which tissue changes. If the lens is small, buffering may be difficult, resulting in a relatively large range of displacement, leading to anterior dislocation of the ciliary ring and even malignant glaucoma (29). In the present study, average light sensitivity in the visual field examination, which provides a quantitative measure of visual function, was a useful parameter in the construction of the prediction model. As can be seen in **Table 1**, the overall light sensitivity of the glaucoma patients was poor, with light sensitivity in the ineffective cohort lower than that in the effective cohort. This finding may be due to severe optic neuropathy caused by high IOP and progressive loss of visual function, which increases the risk of postoperative fundus hemorrhage, resulting in unsatisfactory surgical results (30, 31).

We used specific demographic and disease-related parameters to develop a relatively accurate nomogram for IOP prediction (32). As indicated by the C-index (0.910), the prediction results were consistent with the actual results, and the prediction accuracy was high. The external validation of the cohort revealed a C-index of > 0.9, which pointed to high accuracy of the prediction model. However, the C-index does not take account of the benefits and risks associated with different cut-off points of the model. Therefore, we drew a decision curve to meet the actual needs of clinical decision making and improve the insight of the clinical results.

In this study, we constructed an individualized prediction model to shed light on the factors affecting IOP lowering in glaucoma patients and aid surgical patient selection. In glaucoma patients, when the anterior chamber angle is completely closed, it is difficult to reconstruct the aqueous channel. Extensive iridectomy can relieve the symptoms, and a vitrectomy combined with a lensectomy can unblock the aqueous humor drainage path (33, 34). For younger glaucoma patients, postoperative anti-inflammatory treatments, including anti-inflammatory and anti-infective drugs, should be considered, as these not only relieve ciliary body edema but also protect the optic nerve (35). Persistently high IOP prior to surgery increases the probability of surgical failure in terms of IOP lowering, with the risk of failure increasing in accordance with the duration of IOP elevation. Therefore, we must strive for the best time of operation and try our best to perform the operation under the condition of effectively controlling IOP. In cases where the IOP cannot be lowered *via* a trabeculectomy, hypotensive medication may be used to increase aqueous humor outflow. Ultrasound cycloplasty is another treatment option that has been applied in the clinic in recent years. The aim of the latter is to achieve the best therapeutic outcome by reducing the IOP slowly to the maximum extent (36–38). It is difficult to effectively improve the preoperative axial length and visual field MS, but it can prompt doctors and patients that the operation effect may be poor to allow them to choose other treatment methods or make psychological preparation for the prognosis.

Several limitations of the study should be mentioned. First, All the clinical parameters included in the model selected were based on the characteristics of patients who presented to the ophthalmology department of two hospital (39). The findings of the predictive model were not validated in patient cohorts/populations from other regions or countries, and clinical multicenter trials can be used in the future to increase promotion. To improve the utility of our predictive model, the findings need to be validated in other populations and countries (40). Second, the follow-up time of this study was short, and most of the patients were not observed in the hospital for a long time after the operation due to travel distances from the hospital or other reasons. Therefore, the results of the present study are applicable only to IOP in the early postoperative period, and the long-term effect of a trabeculectomy on IOP needs to be further explored. We hope that more patients can be followed up regularly and long-term in order to build a more perfect prediction model.

## Conclusion

This study presents a new, easy to use nomogram, with high accuracy can be used for individualized prediction of IOP in the early period after a trabeculectomy. The nomogram can help ophthalmologists to assess the risk that IOP will not fall to the normal range in glaucoma patients after the surgery, thereby improving treatment planning and enabling effective, timely intervention measures to be taken to avoid treatment delays or inappropriate treatment. In the past studies, there were no specific prediction studies on intraocular pressure after trabeculectomy. Therefore, the model is a predictive method worthy of our expectation and can hopefully guide clinical practice.

## Data availability statement

The data analyzed in this study is subject to the following licenses/restrictions: Patients with medical record. Requests to access these datasets should be directed to liningnature@126.com.

## Ethics statement

Written informed consent was obtained from the individual(s) for the publication of any potentially identifiable images or data included in this article.

## Author contributions

RW: Conceptualization, Methodology, Formal analysis, Writing - Original Draft, Data Curation, Visualization. NL: Investigation, Validation. YT: Investigation, Data Curation. XC: Validation, Writing - Review, Supervision, Project administration, Funding acquisition. All authors contributed to the article and approved the submitted version.

## Funding

This study was funded by the Jiangsu Province Youth Medical Key Talents (QNRC2016364), the National Natural Science Foundation of China (No. 81500716) and the Anhui Provincial Natural Science Foundation (1408085QH158).

## Conflict of interest

The authors declare that the research was conducted in the absence of any commercial or financial relationships that could be construed as a potential conflict of interest.

Copyright © 2022 Ruixue Wang et al. This is an open access article distributed under the Creative Commons Attribution License, which permits unrestricted use, distribution, and reproduction in any medium, provided the original work is properly cited.

## Publisher’s note

All claims expressed in this article are solely those of the authors and do not necessarily represent those of their affiliated organizations, or those of the publisher, the editors and the reviewers. Any product that may be evaluated in this article, or claim that may be made by its manufacturer, is not guaranteed or endorsed by the publisher.
